# Hippocampal vascular supply and its mediating role in systemic physiological influences on hippocampal volume

**DOI:** 10.3389/fnagi.2025.1590242

**Published:** 2025-08-06

**Authors:** Tae Kim, Javier Rasero, Anna L. Marsland, Mark R. Scudder, Tamer S. Ibrahim, Peter J. Gianaros

**Affiliations:** ^1^Department of Radiology, University of Pittsburgh, Pittsburgh, PA, United States; ^2^Department of Bioengineering, University of Pittsburgh, Pittsburgh, PA, United States; ^3^School of Data Science, University of Virginia, Charlottesville, VA, United States; ^4^Department of Psychology, University of Pittsburgh, Pittsburgh, PA, United States; ^5^Department of Psychiatry, University of Pittsburgh, Pittsburgh, PA, United States

**Keywords:** hippocampus volume, cerebrovascular health, hippocampal vessel segmentation, cardiovascular risk, autonomic nervous system, vascular inflammation, lateralization

## Abstract

**Background:**

Aging-related systemic cardiovascular changes can impair cerebrovascular circulation, contributing to hippocampal atrophy and cognitive decline. However, the mechanistic pathways by which systemic alterations may relate to hippocampal atrophy via hippocampal vascular features remain unclear.

**Methods:**

In this study, 191 participants (aged 30–59 years, 115 female) underwent 7T MRI to segment hippocampal supply vessels and hippocampal volume from T1-weighted images. Twenty-three systemic parameters related to the metabolic syndrome, autonomic function, inflammation, vascular stiffness, and endothelial function were measured at rest. Mediation analysis examined whether hippocampal vessel velocity and size mediated the relationship between systemic factors and hippocampal volume.

**Results:**

Hippocampal volume was highly associated with hippocampal supply vessel velocity, showing a pronounced right lateralized effect. Indirect associations of vessel velocity with hippocampal volume were identified for circulating vascular and intercellular adhesion molecules, heart rate variability, fasting insulin, and spontaneous baroreflex sensitivity. No significant mediated relationships were found for blood pressure, adiposity, mean heart rate, cardiac output, pre-ejection period, reactive hyperemia, pulse wave velocity, mean carotid artery intimal medial thickness, fasting glucose, lipid levels, circulating interleukin-6, hemoglobin A1C, or blood pressure variability.

**Conclusion:**

These findings highlight the role of vascular inflammation, autonomic dysfunction, and metabolic disturbances in hippocampal atrophy, with hippocampal vessel velocity serving as a key mediator. This insight advances our understanding of cerebrovascular contributions to hippocampal structural integrity and cognitive health.

## 1 Introduction

The hippocampus plays an essential role in learning, memory and spatial information processing. However, it is especially vulnerable to hypoxic and ischemic injury compared to other brain regions, and it is one of the first regions in the brain to show alterations in neurodegenerative diseases (Anand and Dhikav, 2012; Johnson, 2023; Mu and Gage, 2011; Nikonenko et al., 2009; Zhang et al., 2022). A reduction in hippocampal volume is often considered as a hallmark of these diseases (Dubois et al., 2010; Mueller et al., 2010). Since maintaining cerebrovascular health is crucial for preserving brain tissue function and integrity, alterations in the blood supply to the hippocampus may contribute to hippocampal atrophy and risk for neurocognitive decline (Johnson, 2023; Brown and Thore, 2011; Perosa et al., 2020; Chao et al., 2010).

The brain is passively perfused by blood flow throughout cardiac pulsation to maintain its metabolism. Thus, persistent exposure to alterations in the cardiovascular system and systemic circulation can progressively impair cerebrovascular circulation, potentially contributing to brain tissue volume loss and cognitive decline, particularly in aging adults (Song et al., 2020). Studies have shown that systemic alterations, including cardiovascular risks, can disrupt cerebral perfusion and are associated with regional brain atrophy (e.g., hippocampus) and cognitive decline (Song et al., 2020; Jefferson et al., 2010; Debette et al., 2011). However, the mechanistic pathways by which systemic conditions influence vessel health in the brain, as well as their specific effects on the hippocampus, remain poorly understood. Unraveling the pathways linking systemic alterations to hippocampal atrophy via its vascular supply could provide insights into cardiovascular and cerebrovascular aging and disease.

Despite the importance of hippocampal vasculatures, neuroimaging studies have rarely explored their functional and structural features compared to the wealth of brain imaging studies of hippocampus morphology and activity. Recently, however, the anatomical characteristics of the hippocampal vascular supply patterns were noninvasively identified by high-resolution MR angiography at high magnetic field [e.g., 7 Tesla (T)] (Perosa et al., 2020; Spallazzi et al., 2019). This method exploits the unsaturated longitudinal magnetization of the flowing blood entering the measurement volume, leading signal intensities to be much higher than the saturated stationary brain tissue with RF pulses. This phenomenon also could be observed with conventional T1-weighted MPRAGE images at 7T (Madai et al., 2015; Maderwald et al., 2008; Zwanenburg et al., 2008). Accordingly, it is now methodologically possible to study hippocampal vasculature features and extend prior neuroimaging studies of hippocampus morphology and activity.

Here, we developed a method to segment blood vessels from T1-weighted images to assess the size and velocity of hippocampal supply vessels, capturing vascular features that have not been previously measured. This approach may provide for a comprehensive characterization of the structural and functional properties of hippocampal blood supply and its potential relationship with hippocampal atrophy. Additionally, since the left and right hippocampi are supplied by separate vascular systems, cardiovascular risk factors may uniquely impact each hemisphere. In this study, we tested whether hippocampal vascular features statistically account for (mediate) any observed relationships between various systemic cardiovascular, metabolic, and autonomic variables and hippocampal volumes in each hemisphere.

## 2 Materials and methods

### 2.1 Participants

A total of 191 participants (aged 30–59 years, mean age 44.9 ± 8.5 years, 115 female and 76 male) comprised the present study sample. These participants were drawn from the Neurobiology of Adult Health (NOAH) study. NOAH participants included midlife men and women who resided in the greater Pittsburgh, Pennsylvania (United States) region at the time of testing. Recruitment methods and related sampling details for the NOAH cohort have been published (Rasero et al., 2025). In brief, participants were recruited via (a) mass electronic and print mailings to residents of Allegheny County, Pennsylvania, United States; (b) radio, electronic (e.g., Social Media, Craigslist, etc.), and print advertisements in public places (e.g., buses, local newspapers, community and park announcement boards); and (c) direct solicitation from the participant registries of the University of Pittsburgh’s Clinical and Translational Science Institute Pitt+Me Registry and University Center for Social and Urban Research Regional Research Registry. Exclusion criteria were: (a) self-reported use of antihypertensive or cardiac medications, anticonvulsants, anti-Parkinson medications, anti-HIV medications, psychotropics, insulin, chemotherapy agents, immunosuppressants and related biological agents, prescription weight-loss medications, or ephedrine (over the counter); regular use of sleep medications, asthma oral and inhalant medications, glucocorticoids, medical marijuana, or more than two non-insulin medications for diabetes; (b) self-reported consumption of ≥ 35 alcoholic drinks in the past 7 days (Gianaros et al., 2023), which may impact the cardiovascular system and cerebrovasculature and potentially confounding the interpretation of study findings; (c) self-reported chronic medical conditions; (d) self-reported history of a major neurological disorder or brain injury resulting in ongoing symptoms or cognitive impairment; (e) lung disease requiring regular or ongoing drug treatment; (f) weight loss surgery within the past 5 years; (g) pregnancy among women; (h) regular use of an assistive walking device; (i) non-fluency in English; (j) night shift employment on a frequent basis; (k) lack of reliable access to a telephone throughout the day - the study required eight visits, including a 7 days ambulatory monitoring and ecological momentary assessment protocol, with daily end-of-day phone interviews conducted by an experimenter. Participants were also excluded for MRI contraindications. Participants were compensated up to $50.00 United States for completing the 7T portion of the NOAH study. Informed consent was obtained from all study participants, and study approval was granted by The University of Pittsburgh Human Research Protection Office.

### 2.2 General study procedures

At the time of initial testing, NOAH participants attended multiple study visits. These visits entailed (a) informed consent; (b) medical history and demographic interviewing; (c) anthropometric assessments of height, weight, and body composition; (d) seated assessments of blood pressure; (e) completion of questionnaires to assess health behaviors and psychosocial characteristics; (f) carotid artery ultrasound; (g) fasting phlebotomy; (h) assessments of cardiovascular and autonomic risk factors; (i) cognitive screening; and (j) MRI protocols. The present study reports imaging data obtained using a 7T Magnetom system (Siemens Inc, Erlangen, Germany), equipped with the 1^*st*^ and 2^*nd*^ generation Tic-Tac-Toe RF head coil, which tend to provide highly consistent homogeneous field excitation across subjects (Krishnamurthy et al., 2019; Santini et al., 2021; Santini et al., 2018; Sajewski et al., 2023).

### 2.3 Systemic variables

A total of 23 systemic parameters of physiology and cardiometabolic risk were explored as factors explaining inter-individual variability in hippocampal vascular features and tissue volume. These encompassed features of the metabolic syndrome, autonomic function, systemic inflammation, vascular stiffness and carotid artery morphology, and endothelial activity. Specific parameters included: systolic blood pressure (SBP); diastolic blood pressure (DBP); waist circumference; body mass index (BMI); mean resting heart rate (HR); the standard deviation of normal-normal beats (SDNN); natural log-transformed variance of high-frequency (0.15–0.4 Hz) heart rate variability (HF-HRV); cardiac output (CO); pre-ejection period (PEP); reactive hyperemia, as reflected by the percentage increase in forearm blood flow following venous occlusion (VOP%); pulse wave velocity (PWV); mean carotid artery intimal medial thickness (IMT); fasting levels of glucose, insulin, low density lipoproteins (LDL), very-low density lipoproteins (vLDL), circulating interleukin-6 (IL-6), plasma intercellular adhesion molecule (ICAM-1), plasma vascular cellular adhesion molecule (VCAM-1), and hemoglobin A1C; the standard deviation of beat-to-beat SBP (SBP_SD); low-frequency variance (0.04–0.149 Hz) in beat-to-beat blood pressure variability (LF-BPV); and mean spontaneous baroreflex sensitivity (BRS). Methodological details for the assessment of these parameters are provided in the Supplementary material.

### 2.4 Hippocampal segmentation

Anatomical 3D T_1_-weighted images (Magnetization-prepared rapid gradient-echo, MPRAGE) were acquired with TR/TE = 3,000/2.1 ms, TI = 1.2 s, FOV = 172 × 240, matrix = 230 × 320, number of slices = 256, voxel-size = 0.75 mm isotropic, acceleration factor (GRAPPA) = 2, TA = 5:02. The volume segmentation for each brain region including left and right hippocampus was performed with Freesurfer 6.0^1^ after bias field correction, and removal of gradient nonlinearity and readout distortion (Glasser et al., 2013). Intracranial volume (ICV) was also obtained from the Freesurfer parcellation procedure.

### 2.5 Hippocampal blood vessel segmentation

For each subject, thin tubular structures of blood vessels were enhanced using a 3D Haar transform following signal intensity normalization (Hou et al., 2017). Briefly, eight neighboring image cubes were extracted from a small 3 × 3 × 3 neighborhood around the center of the reference cube. All the extracted cubes were a group of 7 × 7 × 7 cubes. A Haar transform was applied across these cubes using an 8 × 8 Haar transform matrix. Image enhancement was achieved by the enhanced Haar transform coefficient (C_*En*_)


$$C_{E\,n}\,\left\{ \begin{array}{c} C_{H},i\,f\,\left\lfloor C_{H}\right\rfloor >\alpha_{1}\\ \gamma_{1}\,C_{H},i\,f\,\alpha_{2}\leq \left\lfloor C_{H}\right\rfloor \leq \alpha_{1}\\ \gamma_{2}\,C_{H},i\,f\,\beta \leq \left\lfloor C_{H}\right\rfloor \leq \alpha_{2}\\ 0,i\,f\,\left\lfloor C_{H}\right\rfloor \leq \beta \end{array}\right.$$

, where C_*H*_ is the computed Haar transform coefficient, and α_1_, α_2_ and β are the thresholds to determine the Haar transform coefficient corresponding to image intensity boundary for normal, weak image and noise, respectively. Amplifying factors (γ_1_ and γ_2_) were applied with parameters of α_1_ = 60, α_2_ = 30, β = 10, γ_1_ = 40 and γ_2_ = 0.5⋅γ_1_, identified by varying the parameters. The Frangi vesselness filter, an algorithm to detect tube-like structures, was then applied (Frangi et al., 1998). The vessel segmentation was refined by excluding white matter (WM), cerebellum, temporal, superiormarginal and orbitofrontal regions using a threshold of greater than 5% of the results. Segmented vessels connected to the hippocampus were identified for both hemispheres and then visually inspected for all subjects. Voxel counts of these vessels were calculated as the “hippocampal supply vessels volume” (i.e., the volume of vessels supplying the hippocampus).

To standardize signal intensity (SI) of the segmented hippocampal vessels across subjects, the T1w image signal intensity was normalized to the mean of the WM mask (Reinhold et al., 2019), where only voxels with 100% probability were included (segmentation using FSL’s FAST) to minimize partial volume from other tissues. Normalized SI in the hippocampal supply vessel regions was averaged and reported as “hippocampal supply vessel SI.” The lateralization indices (LI) for hippocampal volume, hippocampal supply vessels volume, and hippocampal supply vessel SI were calculated as LI = (right−left)/(right + left) (Binder et al., 1996; Desmond et al., 1995).

### 2.6 Relationships between systemic variables and hippocampal volume via hippocampal supply vessel

A mediation (path) analysis was performed using the SEM module in JASP^2^, which is based on the Lavaan package of *R* (Rosseel, 2012), to evaluate the relationship between each systemic metric and hippocampal volume (both hemispheres, as well as left and right separately) through the hippocampal supply vessel. A total of 138 mediation models were tested, comprising 23 systemic variables × 3 hippocampal volume measures (total, left and right) × hippocampus supply vessel volume and SI. Fasting insulin and plasma-IL6, which exhibited skewed distributions, were log-transformed prior to analysis, with 1 added before the transformation. The null hypothesis of zero indirect, direct, and total effects was tested in the mediation analysis using 95% bootstrap confidence intervals (CIs) generated from 5,000 iterations. Age, sex, and ICV were included as covariates in the analysis. Statistical significance was determined when the null hypothesis (e.g., indirect effect = 0) fell outside the 95% CI. Unlike the typical *p*-value, which assumes a normal sampling distribution, the bootstrap CI is based on the 2.5^th^ and 97.5^th^ percentiles of the empirical non-parametric bootstrap distribution. The distribution for indirect effects is usually not normally distributed. Since CI does not require an assumption of normality, this approach is more reliable (Flechner and Tseng, 2011; Hayes and Scharkow, 2013). For additional rigor, statistically significant results were re-tested with 50,000 bootstrap iterations to confirm the findings.

## 3 Results

### 3.1 Segmentation of vessels supplying the hippocampus

**FIGURE 1:**
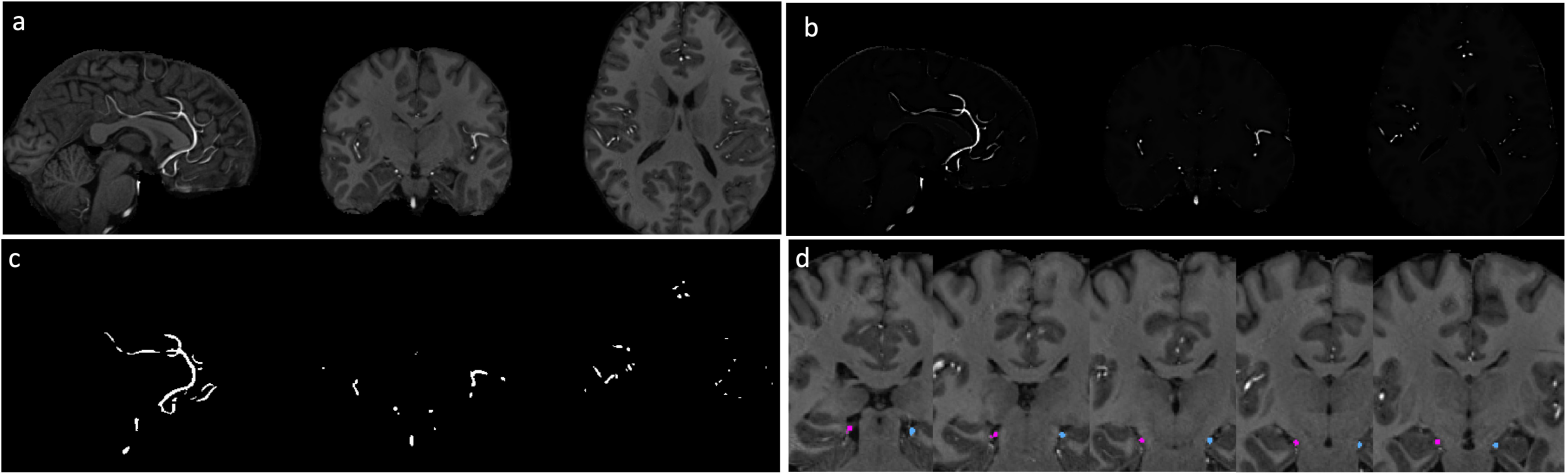
Segmentation of hippocampal vessels. **(a)** MPRAGE T1w images at 7T. **(b)** Vessels are enhanced by 3D Haar transformation. **(c)** The vessels are segmented with Frangi filter. **(d)** Vessels connected with hippocampus were identified. Pink: right hippocampal vessel, cyan: left hippocampal vessel.

Figure 1a demonstrates the high relative signal intensity of arterial vessels with conventional T1w images at 7T. The Haar transformation substantially amplified vessel signals while suppressing non-vascular brain regions (Figure 1b). Segmentation of brain vessels was achieved using the Frangi vesselness filter (Figure 1c). The vessels connecting to the hippocampus, segregated into left and right hemispheres, were successfully identified for all subjects. Vessel segmentation was visually inspected for each subject by overlaying the segmentation results onto the T1w image to ensure accuracy. Figure 1d displays the segmented vessels, with pink and cyan masks overlaid on the T1w image, representing vessels supplying the left and right hippocampus, respectively.

### 3.2 Hippocampal asymmetry

**FIGURE 2:**
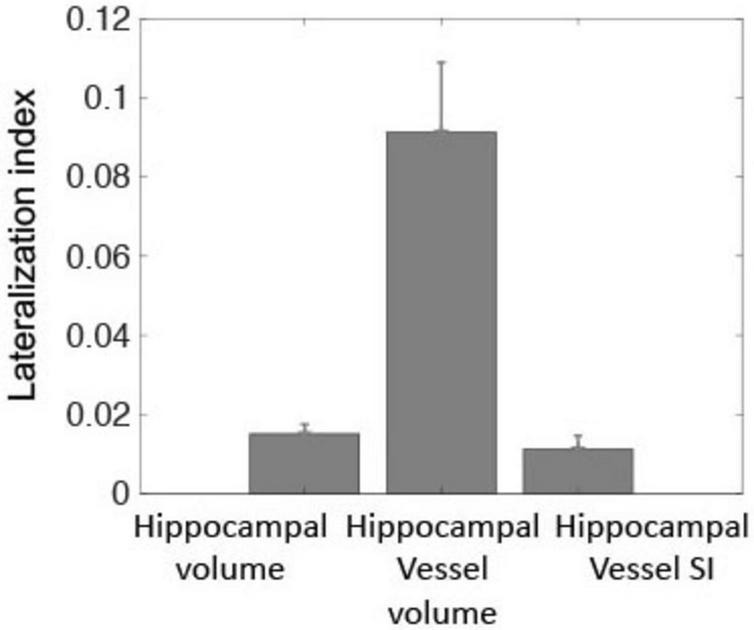
Lateralization indexes were statistically significant, differing from zero for hippocampal volume, hippocampal supply vessels volume, and hippocampal supply vessel SI (*p* < 0.001). These three metrics indicated greater laterality for the right hemisphere relative to the left hemisphere.

The right hippocampal volume was significantly larger than the left [3,730 ± 408 vs. 3,620 ± 403 mm^3^, paired-sample *t*-test, t(190) = 6.1, *p* < 10^–8^], consistent with previous findings on hippocampal volume asymmetry (Pedraza et al., 2004). Similarly, hippocampal supply vessels volume and SI in the right hemisphere was larger than in the left [224 ± 108 vs. 190 ± 91 mm^3^, paired-sample *t*-test, t(190) = 6.1, *p* < 10^–8^ and 1.64 ± 0.27 vs. 1.61 ± 0.29 a.u., t(190) = 2.9, *p* = 0.004, respectively]. Figure 2 shows the lateralization indexes for hippocampal volume, hippocampal supply vessel volume, and hippocampal supply vessel SI, all of which significantly deviated from the null hypothesis (*p* < 0.001).

### 3.3 Relationship between hippocampal supply vessels and hippocampal volume

Intracranial volume (head size) was strongly correlated with hippocampal volume (r = 0.67, *p* = 3.2 × 10^–26^), and was further associated with hippocampal supply vessel volume (r = 0.39, *p* = 3.1 × 10^–8^). Men exhibited larger ICV and hippocampal volumes compared to women (*p* = 3.7 × 10^–21^ and *p* = 6.7 × 10^–11^, respectively), consistent with prior studies (Pedraza et al., 2004; Buckner et al., 2004). Age was significantly correlated with hippocampal volumes after controlling for sex and ICV (r = −0.15, *p* = 0.03), while sex was not significantly correlated with hippocampal volumes after controlling for age and ICV. Consequently, age, sex, and ICV were included as covariates in linear regression models.

**FIGURE 3:**
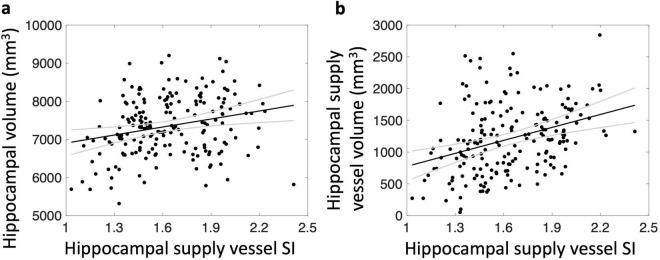
Relationship between hippocampal supply vessel SI and hippocampal volume **(a)** and hippocampal supply vessel volume **(b)**. Hippocampal supply vessel SI are associated with hippocampal volumes (r = 0.25, *p* < 0.01) **(a)** and hippocampal supply vessel volume (r = 0.35, *p* < 0.01) **(b)**

After adjusting for covariates, total (= left + right) hippocampal supply vessel volume was not significantly associated with hippocampal volume (*p* = 0.19). However, total hippocampal supply vessel SI was positively correlated with total hippocampal volume (r = 0.25, *p* = 0.007) (Figure 3a). Significant associations were also observed between left and right hippocampal supply vessel SI and their respective hippocampal volumes (r = 0.26, *p* = 0.008 and r = 0.20, *p* = 0.03, respectively). Additionally, the hippocampal supply vessel SI was highly associated with hippocampal supply vessel volume (r = 0.35, *p* = 1.9 × 10^–5^) (Figure 3b).

### 3.4 Relationship between systemic metrics and hippocampal volume via hippocampal supply vessel SI

**FIGURE 4:**
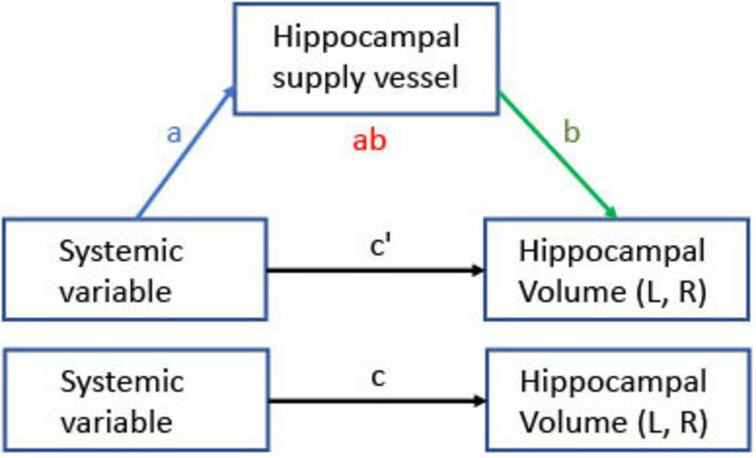
Mediation model for relationships between systemic variables and hippocampal volume through hippocampal supply vessel SI. Arrows indicate effects; a is the effect of a systemic variable on the hippocampal vessel SI, b is the effect of the hippocampal vessel SI on hippocampal volume, the ab product is the indirect effect of a systemic variable on hippocampal volume through hippocampal supply vessel SI, and c’ is the direct effect. The total effect is the sum of the direct and the indirect effect (c = c’ + a⋅b). Hippocampal volume and supply vessel SI were separately tested for total, left, and right, respectively.

Figure 4 depicts the mediation model framework examining the relationships between each systemic factor and hippocampal volumes (left, right, and total) mediated by hippocampal supply vessel SI (left, right, and total, respectively). Arrows in Figure 4 represent the following effects: c denotes the total effect of the systemic variable (X) on hippocampal volume (Y), corresponding to the sum of the direct and indirect effects. The product of the mediator paths (a⋅b) indicates the indirect (mediated) effect. Lastly, c’ denotes the effect of X on Y after controlling for mediators (c’ = c−a⋅b). Table 1 summarizes the statistically significant pathways in the mediation models. Estimated coefficients, *p*-values, and CI are provided in Supplementary Table 1. The mediation model of hippocampal supply vessel volume did not show any significant paths except for the path from insulin to total hippocampal supply volume (Supplementary Table 2)

**TABLE 1:** Mediation analysis results linking systemic variables to hippocampal volume via hippocampal supply vessel signal intensity (SI).

	Path a				Path b				Path a⋅b			
Plasma VCAM	T	L	R	−	T	L	R	+	T	L	R	−
Plasma ICAM	T	L	R	−	T	L	NS	+	T	L	NS	−
SDNN	T	L	R	+	T	L	NS	+	T	L	R	+
HF HRV	T	NS	R	+	T	L	NS	+	T	NS	NS	+
Average BRS	T	L	R	+	T	L	NS	+	T	L	R	+
Insulin	NS	L	NS	−	NS	L	NS	+	NS	L	NS	−
CO	NS	L	NS	+	NS	L	NS	+	NS	NS	NS	

T, L, and R indicate statistically significant effects for total (= left + right), left, and right hippocampal volume, respectively. All c’ and c paths were non-significant and therefore not included in the table. +, positive; −, negative relationship; NS, statistically not significant.

Plasma VCAM-1, plasma ICAM-1, SDNN, HF HRV, and average BRS showed significant mediating relationships with total hippocampal volume through total hippocampal vessel SI. Regarding hemispheric laterality, plasma VCAM-1, SDNN, and average BRS showed a significant effect on both hemispheres, while plasma ICAM-1 and HF HRV showed effects for the left hemisphere. Insulin showed an indirect effect exclusively on left hippocampal volume via left hippocampal vessel SI.

## 4 Discussion

The development of a cerebral vessel segmentation technique successfully extracted hippocampal supply vessel signals from high-resolution T1-weighted images at 7T. This approach allowed us to evaluate the influence of hippocampal supply vessels on the relationships between systemic variables and hippocampal volume. Our findings revealed that plasma VCAM-1, plasma ICAM-1, SDNN, HF-HRV, fasting insulin, and average BRS showed an indirect association with hippocampal volume through hippocampal supply vessel SI, without evidence of a direct and total relationship. This suggests that hippocampal supply vessel SI may mediate the effects of these variables on changes in hippocampal volume.

### 4.1 Hippocampal supply vessel

In this study, we leveraged the large image contrast between fresh inflowing blood and stationary tissue in high-resolution MPRAGE images at 7T to detect vessel signals. T1-weighted MPRAGE employs a non-slice selective inversion recovery (IR) pulse. While lower magnetic field systems (e.g., 3T or 1.5T) typically use a body coil for RF transmission and a local head coil for signal reception, 7T MRI utilizes a head-coil for both transmission and reception. At 7T, the non-slice selective IR pulse inverts spin only within the head coil’s coverage, resulting in the slab selective inversion. Consequently, blood entering from outside the head coil is unaffected by the IR pulse, while stationary tissues are affected by the RF pulse. Longer longitudinal relaxation time at 7T also leads to faster saturation of stationary tissue, enhancing contrast between tissue and fresh inflowing blood. As a result, blood appears brighter than other tissues and fluids in MPRAGE images, providing remarkable depiction of vascular structure. In addition, the signal intensity of fresh blood spins can vary with velocity, as faster-moving blood is less susceptible to RF pulses within the imaging volume, compared to slower-moving blood at a given location. Thus, hippocampal vessel SI may reflect blood velocity, with faster flow producing higher signals.

The optimized MRI angiography (MRA) approach using T1w-MPRAGE (MPRAGE-MRA) has been assessed against standard MRA methods (Madai et al., 2015; Maderwald et al., 2008; Zwanenburg et al., 2008). Superior depiction of small vessel structures was demonstrated at 7T compared to 3T TOF MRA in cerebrovascular disease (Madai et al., 2015). A comparison of MRAs from three different gradient echo sequences (TOF, VIBE, and MPRAGE) showed that the MPRAGE approach was superior for vessel depiction in general 3D image, while TOF performed best fit for maximum intensity projection (MIP) images at 7T (Maderwald et al., 2008). MPRAGE-MRA captures whole-brain coverage and combines tissue and vascular anatomy in a single scan, whereas TOF MRA generally could require more acquisition time due to specific absorption rate (SAR) constraints and is thus more suited for maximum intensity projection (MIP) images. These advantages position 7T MPRAGE-MRA as an effective tool for visualizing vascular structures, including hippocampal supply vessels.

### 4.2 Hippocampal volume and hippocampal vascular supply

Hippocampal volume is closely linked to cognition (Van Petten, 2004). Aging is associated with reduced hippocampal tissue volumes, as well as alterations in its vascular supply. Our findings demonstrate a strong correlation between hippocampal volume and its vascular supply, a relationship supported by prior studies (Johnson, 2023; Perosa et al., 2020; Spallazzi et al., 2019). The structural and functional changes of the hippocampal vasculature, regarded as part of the vascular reserve of the hippocampus, may be a primary contributing factor to hippocampal volume changes (e.g., atrophy), and this relationship therefore may play a role in cognitive decline.

In addition, hippocampal asymmetry, where the right hippocampus is typically larger in volume than the left (Pedraza et al., 2004), may be partially explained by a greater vascular supply to the right side. Our findings showed consistent lateralization indices for hippocampal volume and vascular supply, suggesting that a larger right hippocampus may require greater blood flow. This asymmetrical vascular supply could contribute to hemisphere-specific vulnerabilities, potentially contributing to functional declines that varied by hemisphere. For example, verbal memory decline is associated with left hippocampal atrophy, and left hippocampal dysfunction is observed earlier than the right (Baxter et al., 2023). A lower vascular supply to the left hippocampus may increase its vulnerability to pathological processes and could be associated with the left-predominant atrophy pattern characteristic of Alzheimer’s disease progression (Cherbuin et al., 2010). Therefore, maintaining hippocampal vascular health may be critical for preserving neurocognitive function.

### 4.3 Systemic variables and hippocampus relationships

Systemic physiological changes may influence cerebrovascular structure and function, affecting hippocampal health. Plasma VCAM-1 and ICAM-1 demonstrated indirect relationships with hippocampal volume through vessel velocity. VCAM-1 is thought to reflect endothelial activation and chronic inflammation, suggesting that it may impact vascular dysfunction and reduced perfusion (Tchalla et al., 2017), which can be critical mediators of hippocampal atrophy. Plasma ICAM-1, also implicated in vascular inflammation, was further observed in the present study to relate indirectly to volume in both hemispheres, with a stronger influence on the left, indicating lateralized vulnerability.

Autonomic physiology, as reflected by measures such as SDNN, HF-HRV, and BRS, also showed indirect effects on hippocampal volume via vessel velocity. Reduced SDNN and BRS may reflect impaired autonomic control over cardiovascular function, which may influence cerebrovascular dynamics such as vascular tone and blood flow regulation. Indeed, some evidence suggests that such measures relate to cognition and dementia risk, possibly via neurovascular pathways (Liu et al., 2022; Magnon et al., 2022; Ma et al., 2025). Autonomic dysregulation can also be indirectly influenced by inflammation, stress, and metabolic dysfunction (Hu et al., 2018; Marsland et al., 2007; Rosano et al., 2012). HF-HRV, a component of heart rate variability that is thought to predominantly reflect parasympathetic cardiac activity, cardiorespiratory function, and cardioventilatory integration (Grossman, 2024), showed a stronger association with the left hippocampus, consistent with previous findings that indicators of parasympathetic nervous system activity may exhibit leftward cerebral lateralization, while those reflecting sympathetic activity may be right-lateralized (Ferraro et al., 2022; Hilz et al., 2001).

Insulin is known to influence vascular function by enhancing arterial compliance, relaxing resistance arterioles to improve blood flow, and dilating precapillary arterioles to increase microvascular blood volume (Zheng and Liu, 2015). Insulin exhibited an indirect effect on left hippocampal volume. The left hippocampus may be more vulnerable to disruptions in vascular function caused by metabolic alterations such as insulin resistance, emphasizing the interplay between metabolic and vascular health.

Interestingly, systemic parameters such as BMI, blood pressure (systolic, diastolic, and variability measures), lipid profiles (LDL and vLDL), glucose levels, and vascular measures (IMT and PWV) did not show significant pathways to hippocampal volume through hippocampal vessel velocity. This suggests that local factors, including autoregulation and regional metabolic demand in the brain, may exert a stronger influence on hippocampal vascular dynamics than global systemic measures. The vessel size assessed in systemic and cerebrovascular (macrovascular vs. microvascular) vessels may also be a contributing factor. Furthermore, systemic vascular changes may not always directly translate to localized cerebrovascular alterations, particularly in the context of the temporal gap between systemic changes and their cerebrovascular manifestations or compromised brain regulation under pathological conditions.

Taken together, our findings suggest that the multiple pathways, including systemic vascular inflammation, autonomic dysfunction, and metabolic disturbances, contribute to hippocampal structural integrity, with hippocampal supply vessel velocity serving as a critical mediator. This interplay may underscore the complex mechanisms underlying hippocampal atrophy and its potential implications for cognitive health. The lower hippocampal vascular density relative to its high metabolic demand may make it particularly vulnerable to conditions of reduced vascular function (Johnson, 2023). This highlights the importance of maintaining cerebrovascular health in mitigating hippocampal atrophy and its potential cognitive consequences. The systemic factors that relate to cerebrovascular-mediated pathways are potentially modifiable by therapeutic and preventative interventions. Understanding these mechanisms could lead to novel strategies to reduce the risk of cognitive decline and cerebrovascular diseases.

### 4.4 Limitations and future directions

While we observed age-related reductions in hippocampal volume, our midlife sample was younger than the later life populations typically affected by pronounced hippocampal atrophy. As such, age-related acceleration of atrophy may not be as pronounced in this cohort, and findings may be of limited generalizability to later life adults. However, midlife neurovascular changes may identify those at risk for future cognitive decline, and midlife is a period when preventative efforts may be most effective (Dohm-Hansen et al., 2024). The current study is cross-sectional, limiting the ability to draw causal inferences regarding the impact of cardiovascular alterations on hippocampal atrophy and related cognitive decline in older age. Future studies could include adult cohorts with a broader age range, including those experiencing significant atrophy, to confirm and expand upon the current findings. Longitudinal research will also be essential to clarify the temporal and potentially causal roles of modifiable cardiovascular risk factors in hippocampal degeneration and cognitive decline. Nonetheless, these findings offer preliminary support for a potential cascade mechanism linking systemic, cerebrovascular-mediated factors to hippocampal vulnerability.

## Data Availability

The original contributions presented in this study are included in this article/Supplementary material, further inquiries can be directed to the corresponding author.

## Footnotes

1
https://surfer.nmr.mgh.harvard.edu/

2
https://jasp-stats.org/
