# Supplementary material for: Hippocampal vascular supply and its mediating role in systemic physiological influences on hippocampal volume
Source: Front Aging Neurosci. 2025 Aug 6;17:1590242. doi: 10.3389/fnagi.2025.1590242 (PMC12364891; doi:10.3389/fnagi.2025.1590242)
Supplement: Supplementary file 1 [file Data_Sheet_1.docx]

**Supplementary Materials**

**Methods**

Prior to their laboratory visit, participants were asked to refrain from exercising, consuming a meal, and drinking caffeine for 3 hours. They were also asked to refrain from using nicotine products for 2 hours prior to their visit, as well as drinking alcohol after 9 PM the night before their visit. Lastly, they were asked to refrain from cold, allergy, or headache medications for 12 hours prior to the visit. Participants reporting symptoms consistent with a cold or infection were rescheduled for a different day. Participants were screened to ensure they had followed these instructions, as well as to confirm that they had not taken antibiotics in the past 2 weeks. During the laboratory visit, phlebotomy was performed, and assessments of anthropometrics, BPs, and autonomic and cardiovascular physiology were obtained. To assess cardiovascular and autonomic function, participants were fitted with electrodes on the chest and calf for an electrocardiogram (ECG) and impedance cardiogram (ICG). Carotid artery intima media thickness was measured in a separate visit. Assessment protocols and systemic variables tested as mediators are described as follows:

*Seated resting blood pressure assessments.* Seated brachial BPs were taken at rest with an Omron IntelliSense© BP Monitor (model HEM-907XL, Omron Healthcare Inc.). SBP and DBP values in the present report were computed from the mean of last 2 of 3 readings after an acclimation period in accordance with American Heart Association guidelines (1).

*Anthropometric assessments of adiposity.* Waist circumference (in centimeters) was measured at the point of the iliac crest at end expiration. Body mass index was derived as weight in kg/height [m^2^], with height measured using a vertical-mounted stadiometer.

*Electrocardiographic assessments of heart rate (HR) and heart rate variability (HRV).* Digitized recordings of inter-beat intervals (IBIs) were obtained by a modified 2-lead electrocardiogram (ECG) obtained during a 5-min period of seated rest to assess mean HR and 2 HRV metrics following established guidelines (2). In addition to the mean HR (beats per minute), the SDNN (ms) was computed as the total variance (or standard deviation) of normal-to-normal intervals in the IBI time series. The weighted IBI time series was linearly detrended, mean-centered, and tapered using a Hamming window. Fast Fourier Transforms (FFTs) of each of the 60-second IBI time series from the 5-minute recording period were used to compute spectral power (in milliseconds squared per hertz) in the 0.15 to 0.40 (3) bandwidth. The natural log of the spectral power in this bandwidth was used as the estimate of HF-HRV.

*Dual impedance cardiography (ICG) assessments of cardiac output (CO), pre-ejection period (PEP), and pulse-wave velocity (PWV).* CO, PEP, and PWV were measured by thoracic and calf (ICG and d-ICG) methods that have been previously detailed (3). By these methods, the 2-lead ECG described above was obtained concurrently with a basal ICG (Z0) and the first derivative of the pulsatile ICG change (dZ/dt) using tetrapolar lead configurations that were affixed to the (a) thorax to measure aortic flow onset and (b) calf to measure peripheral muscle blood flow onset following established ICG guidelines (4). Ensemble averaging was used to derive the following: cardiac output (computed by the Kubicek equation); PEP (computed as the interval in milliseconds between the R wave of the ECG and the B-point of the dZ/dt waveform); and PWV (computed in m/s as the distance along the arterial tree from the thorax to the calf (height of the acromioclavicular joint to the midpoint between calf electrodes) divided by the transit time (time between the b-point of the dZ/dt signal from the thorax [aortic] and the onset of the pulse waveform from the calf leads).

*Venous occlusion plethysmography for the assessment of forearm blood flow.* Forearm blood flow (FBF) was measured non-invasively by a post-occlusive reactive hyperemia protocol using venous occlusion strain-gauge plethysmography. FBF was specifically assessed using a modified Sivertsson protocol. For these assessments, an Indium-Gallium strain gauge was applied to the forearm and connected to a Hokanson EC5R monitor. An occlusion cuff was positioned on the upper arm, with the arm suspended at heart level. Another occlusion cuff was positioned on the wrist and inflated to 200mmHg by a Hokanson AG101 air-source device. Thereafter, venous occlusion assessments were made by inflating the upper cuff above venous pressure and assessing consequent forearm blood flow. These measures were used to compute baseline FBF (FBFb). Post-occlusive hyperemia-induced maximal vasodilation (FBFmax) was then assessed by inflating the occlusion cuff for 5 min above SBP. After the rapid deflation of the occluding cuff, venous occlusion measures were obtained to assess maximum reactive hyperemia (FBFmax). The final metric used for analyses was the ratio of FBFmax during reactive hyperemia to FBFb, which was converted to a percentage value.

*Assessment of carotid artery intima media thickness***.** Carotid artery intima-media thickness (C-IMT) was measured by B-mode ultrasound. Carotid scans were performed by registered vascular technologists. Participants underwent a ~35 min scan using an Acuson Antares scanner (Acuson-Siemens, Malvern, PA). B-mode imaging focused on 3 regions: (1) the near and far walls of the distal common carotid (1cm to bulb); (2) the far wall of the carotid bulb (from where the walls of the common carotid are no longer parallel to the flow divider); and (3) the far wall of the first cm of the internal carotid (defined by the edge of the flow divider). For these 3 regions (common, bulb and internal), an image was digitized for scoring by semi-automated edge detection software (Artery Measurement System; Goteborg University, Sweden). The software generated two lines: one along the lumen-intima interface and one along the media-adventitia interface. The distances between the interfaces are measured in 1 cm increments, generating one measurement (in mm) per pixel in each region (~140 total). For each region, the average values were recorded. Mean C-IMT used in the present analyses corresponded to the average of all values in both carotids.

*Phlebotomy assessments.* Participants underwent fasting phlebotomy for the assessment of glucose, insulin, low- (LDL) and very-low (vLDL) density lipoproteins, as well as glycosylated hemoglobin (HbA1c). Blood samples were also used to assess circulating IL-6, VCAM-1, and ICAM-1 levels by Simple Plex assays utilizing the antibody-based Ella™ system (ProteinSimple, Biotechne). This system uses a solid-phase sandwich immunoassay. The standard ranges of assays were 0.7 – 2,652 pg/mL for IL-6, 53.7-83,480 pg/mL for VCAM-1, and 2.01-15,630 pg/mL for ICAM-1. Samples were run in triplicate, and average intra- and inter-plate coefficients of variation (CVs) were 5.1% and 8.8% for IL-6, 2.1% and 11.8% for VCAM-1, and 3.5% and 10.7% for ICAM-1, respectively.

*Beat-to-beat BP assessments*. Beat-to-beat (continuous) BP monitoring was performed using a CNAP® 500 finger cuff (CNSystems, Medizintechnik, AG, Austria) placed on the middle and index fingers of each participant’s left hand. These signals were calibrated to an oscillometric blood pressure recording obtained from the brachial artery of the right arm. Spontaneous baroreflex sensitivity (BRS) in ms/mmHg was computed as described previously by the sequence method (3). The total variability in beat to beat SBP was computed as the SD of all SBPs, and the variance in low-frequency SBP was computed by FFT to determine the spectral power in the 0.04 - 0.149 (4) bandwidth.

References

1. Muntner P, Shimbo D, Carey RM, Charleston JB, Gaillard T, Misra S, et al. Measurement of Blood Pressure in Humans: A Scientific Statement From the American Heart Association. Hypertension. 2019;73(5):e35-e66.

2. Quigley KS, Gianaros PJ, Norman GJ, Jennings JR, Berntson GG, de Geus EJC. Publication guidelines for human heart rate and heart rate variability studies in psychophysiology-Part 1: Physiological underpinnings and foundations of measurement. Psychophysiology. 2024;61(9):e14604.

3. Scudder MR, Jennings JR, DuPont CM, Lockwood KG, Gadagkar SH, Best B, et al. Dual impedance cardiography: An inexpensive and reliable method to assess arterial stiffness. Psychophysiology. 2021;58(7):e13772.

4. Sherwood A, Allen MT, Fahrenberg J, Kelsey RM, Lovallo WR, van Doornen LJ. Methodological guidelines for impedance cardiography. Psychophysiology. 1990;27(1):1-23.

**Supplementary Table 1**. Mediation model for relationships between systemic variable and hippocampal volume through hippocampal supply vessel SI. Bold character indicates statistically significant relationship with 95% confidence intervals; italic indicates statistically significant p-value

| **Both** | label | estimated | p-value | CI. lower | CI. upper |
| --- | --- | --- | --- | --- | --- |
| Plasma VCAM | a | -0.256 | *2.2x10^-4^* | **-0.359** | **-0.138** |
|  | b | 0.158 | *0.004* | **0.031** | **0.278** |
|  | c' | 0.065 | 0.235 | -0.046 | 0.160 |
|  | ab | -0.041 | *0.024* | **-0.083** | **-0.010** |
|  | total (c) | 0.024 | 0.650 | -0.084 | 0.113 |
| Plasma ICAM | a | -0.165 | *0.019* | **-0.326** | **-0.042** |
|  | b | 0.146 | *0.008* | **0.020** | **0.269** |
|  | c' | 0.030 | 0.582 | -0.065 | 0.114 |
|  | ab | -0.024 | 0.078 | **-0.069** | **-0.004** |
|  | total (c) | 0.005 | 0.921 | -0.099 | 0.087 |
| SDNN | a | 0.150 | *0.040* | **0.021** | **0.298** |
|  | b | 0.148 | *0.006* | **0.027** | **0.263** |
|  | c' | -0.022 | 0.694 | -0.125 | 0.065 |
|  | ab | 0.022 | 0.099 | **0.005** | **0.058** |
|  | total (c) | 6.8x10^-4^ | 0.990 | -0.088 | 0.087 |
| HF HRV | a | 0.150 | *0.042* | **0.022** | **0.287** |
|  | b | 0.145 | *0.007* | **0.022** | **0.265** |
|  | c’ | -8.4x10^-4^ | 0.988 | -0.109 | 0.104 |
|  | ab | 0.022 | 0.104 | **0.004** | **0.061** |
|  | total (c) | 0.021 | 0.708 | -0.078 | 0.124 |
| Mean BRS | a | 0.189 | *0.008* | **0.064** | **0.323** |
|  | b | 0.150 | *0.006* | **0.023** | **0.274** |
|  | c’ | -0.025 | 0.645 | -0.134 | 0.072 |
|  | ab | 0.028 | 0.056 | **0.007** | **0.071** |
|  | total (c) | 0.003 | 0.949 | -0.088 | 0.098 |
| Insulin | a | -0.140 | 0.051 | -0.285 | 0.004 |
|  | b | 0.147 | *0.007* | **0.020** | **0.269** |
|  | c’ | -0.020 | 0.708 | -0.120 | 0.074 |
|  | ab | -0.021 | 0.113 | -0.068 | 0.000 |
|  | total (c) | -0.041 | 0.454 | -0.143 | 0.060 |
| CO | a | 0.094 | 0.183 | -0.023 | 0.237 |
|  | b | 0.144 | *0.007* | **0.025** | **0.273** |
|  | c’ | 0.013 | 0.801 | -0.089 | 0.112 |
|  | ab | 0.014 | 0.233 | -0.0005 | 0.052 |
|  | total (c) | 0.027 | 0.615 | -0.073 | 0.129 |
| BMI | a | -0.078 | 0.274 | -0.242 | 0.071 |
|  | b | 0.142 | *0.008* | **0.023** | **0.259** |
|  | c’ | -0.041 | 0.432 | -0.031 | 0.055 |
|  | ab | -0.011 | 0.312 | -0.052 | 0.007 |
|  | total (c) | -0.052 | 0.327 | -0.144 | 0.044 |
| SBP | a | -0.018 | 0.802 | -0.173 | 0.122 |
|  | b | 0.145 | *0.006* | **0.030** | **0.265** |
|  | c’ | 0.016 | 0.768 | -0.104 | 0.124 |
|  | ab | -0.003 | 0.803 | -0.034 | 0.017 |
|  | total (c) | 0.013 | 0.810 | -0.109 | 0.125 |
| DBP | a | -0.055 | 0.440 | -0.200 | 0.067 |
|  | b | 0.142 | *0.008* | **0.026** | **0.259** |
|  | c’ | -0.057 | 0.275 | -0.168 | 0.043 |
|  | ab | -0.008 | 0.459 | -0.041 | 0.007 |
|  | total (c) | -0.065 | 0.223 | -0.177 | 0.043 |
| waist | a | -0.083 | 0.243 | -0.219 | 0.053 |
|  | b | 0.143 | *0.007* | **0.023** | **0.262** |
|  | c’ | -0.024 | 0.655 | -0.115 | 0.066 |
|  | ab | -0.012 | 0.284 | -0.051 | 0.004 |
|  | total (c) | -0.035 | 0.058 | -0.126 | 0.059 |
| glucose | a | -0.063 | 0.395 | -0.193 | 0.099 |
|  | b | 0.147 | *0.006* | **0.023** | **0.261** |
|  | c’ | -0.036 | 0.509 | -0.123 | 0.083 |
|  | ab | -0.009 | 0.417 | -0.040 | 0.011 |
|  | total (c) | -0.045 | 0.414 | -0.132 | 0.080 |
| LDL | a | 0.013 | 0.860 | -0.125 | 0.156 |
|  | b | 0.141 | *0.009* | **0.014** | **0.260** |
|  | c’ | -0.017 | 0.753 | -0.123 | 0.080 |
|  | ab | 0.002 | 0.860 | -0.020 | 0.027 |
|  | total (c) | -0.015 | 0.782 | -0.125 | 0.083 |
| vLDL | a | -0.112 | 0.125 | -0.263 | 0.047 |
|  | b | 0.134 | *0.013* | **0.008** | **0.253** |
|  | c’ | -0.058 | 0.280 | -0.197 | 0.066 |
|  | ab | -0.015 | 0.191 | -0.053 | 0.003 |
|  | total (c) | -0.073 | 0.179 | -0.213 | 0.067 |
| hemoglobinA1C | a | 0.092 | 0.215 | -0.051 | 0.258 |
|  | b | 0.142 | *0.009* | **0.011** | **0.265** |
|  | c’ | 0.0004 | 0.994 | -0.121 | 0.109 |
|  | ab | 0.013 | 0.262 | -0.004 | 0.054 |
|  | total (c) | 0.013 | 0.810 | -0.109 | 0.126 |
| Plasma IL-6 | a | -0.056 | 0.446 | -0.192 | 0.099 |
|  | b | 0.141 | *0.009* | **0.022** | **0.254** |
|  | c’ | -0.046 | 0.391 | -0.161 | 0.058 |
|  | ab | -0.008 | 0.464 | -0.038 | 0.011 |
|  | total (c) | -0.054 | 0.324 | -0.171 | 0.051 |
| Mean IMT | a | 0.048 | 0.598 | -0.100 | 0.212 |
|  | b | 0.143 | *0.008* | **0.027** | **0.268** |
|  | c’ | -0.081 | 0.228 | -0.202 | 0.063 |
|  | ab | 0.007 | 0.605 | -0.011 | 0.038 |
|  | total (c) | -0.074 | 0.279 | -0.195 | 0.073 |
| Mean HR | a | -0.010 | 0.887 | -0.139 | 0.122 |
|  | b | 0.145 | *0.006* | **0.027** | **0.274** |
|  | c’ | 0.044 | 0.412 | -0.057 | 0.135 |
|  | ab | -0.002 | 0.887 | -0.025 | 0.019 |
|  | total (c) | 0.042 | 0.438 | -0.059 | 0.134 |
| PEP | a | -0.020 | 0.783 | -0.167 | 0.121 |
|  | b | 0.144 | *0.007* | **0.028** | **0.265** |
|  | c’ | -0.029 | 0.578 | -0.116 | 0.069 |
|  | ab | -0.003 | 0.784 | -0.029 | 0.017 |
|  | total (c) | -0.032 | 0.549 | -0.128 | 0.071 |
| VOP % | a | 0.080 | 0.269 | -0.021 | 0.208 |
|  | b | 0.147 | *0.006* | **0.019** | **0.269** |
|  | c’ | -0.012 | 0.829 | -0.124 | 0.099 |
|  | ab | 0.012 | 0.305 | -0.002 | 0.046 |
|  | total (c) | 0.0002 | 0.996 | -0.110 | 0.111 |
| PWV | a | 0.060 | 0.073 | -0.058 | 0.186 |
|  | b | 0.146 | *0.006* | **0.026** | **0.270** |
|  | c’ | -0.010 | 0.850 | -0.124 | 0.104 |
|  | ab | 0.009 | 0.431 | -0.006 | 0.040 |
|  | total (c) | -0.001 | 0.979 | -0.119 | 0.115 |
| SBP_SD | a | 0.034 | 0.639 | -0.089 | 0.164 |
|  | b | 0.145 | *0.007* | **0.033** | **0.266** |
|  | c’ | 0.022 | 0.685 | -0.114 | 0.140 |
|  | ab | 0.005 | 0.644 | -0.010 | 0.036 |
|  | total (c) | 0.027 | 0.625 | -0.102 | 0.142 |
| LF_BPV | a | 0.013 | 0.853 | -0.116 | 0.157 |
|  | b | 0.145 | *0.007* | **0.025** | **0.263** |
|  | c’ | 0.048 | 0.363 | -0.067 | 0.148 |
|  | ab | 0.002 | 0.853 | -0.016 | 0.030 |
|  | total (c) | 0.050 | 0.353 | -0.066 | 0.149 |

| **Left** | label | | estimated | p-value | CI. lower | CI. upper |
| --- | --- | --- | --- | --- | --- | --- |
| Plasma VCAM | | a | -0.223 | *0.001* | **-0.329** | **-0.109** |
|  | | b | 0.150 | *0.009* | **0.031** | **0.268** |
|  | | c’ | 0.037 | 0.509 | -0.086 | 0.143 |
|  | | ab | -0.033 | *0.044* | **-0.073** | **-0.008** |
|  | | total (c) | 0.004 | 0.945 | -0.118 | 0.105 |
| Plasma ICAM | | a | -0.157 | *0.027* | **-0.296** | **-0.028** |
|  | | b | 0.150 | *0.008* | **0.027** | **0.267** |
|  | | c’ | 0.052 | 0.353 | -0.046 | 0.150 |
|  | | ab | -0.024 | 0.090 | **-0.063** | **-0.004** |
|  | | total (c) | 0.028 | 0.615 | -0.071 | 0.129 |
| SDNN | | a | 0.132 | 0.073 | **0.008** | **0.273** |
|  | | b | 0.146 | *0.009* | **0.023** | **0.265** |
|  | | c’ | 0.023 | 0.682 | -0.075 | 0.120 |
|  | | ab | 0.019 | 0.139 | **0.002** | **0.054** |
|  | | total (c) | 0.042 | 0.458 | -0.050 | 0.139 |
| HF HRV | | a | 0.128 | 0.085 | -0.012 | 0.266 |
|  | | b | 0.146 | *0.009* | **0.021** | **0.263** |
|  | | c’ | 0.026 | 0.646 | -0.081 | 0.132 |
|  | | ab | 0.019 | 0.150 | 6.1x10^-4^ | 0.056 |
|  | | total (c) | 0.045 | 0.437 | -0.059 | 0.149 |
| Mean BRS | | a | 0.172 | *0.016* | **0.027** | **0.314** |
|  | | b | 0.148 | *0.009* | **0.021** | **0.268** |
|  | | c’ | 0.008 | 0.893 | -0.096 | 0.101 |
|  | | ab | 0.025 | 0.077 | **0.004** | **0.066** |
|  | | total (c) | 0.033 | 0.557 | -0.060 | 0.124 |
| Insulin | | a | -0.145 | *0.045* | **-0.273** | **-0.002** |
|  | | b | 0.157 | *0.005* | **0.038** | **0.278** |
|  | | c’ | -0.006 | 0.912 | -0.105 | 0.096 |
|  | | ab | -0.023 | 0.103 | **-0.063** | **-0.002** |
|  | | total (c) | -0.029 | 0.608 | -0.132 | 0.077 |
| CO | | a | 0.121 | 0.088 | **0.002** | **0.249** |
|  | | b | 0.145 | *0.009* | **0.027** | **0.262** |
|  | | c’ | 0.029 | 0.591 | -0.068 | 0.140 |
|  | | ab | 0.018 | 0.153 | 8x10^-4^ | 0.053 |
|  | | total (c) | 0.047 | 0.395 | -0.052 | 0.162 |
| BMI | | a | -0.057 | 0.427 | -0.213 | 0.083 |
|  | | b | 0.147 | *0.008* | **0.031** | **0.266** |
|  | | c’ | -0.023 | 0.672 | -0.117 | 0.077 |
|  | | ab | -0.008 | 0.446 | -0.044 | 0.009 |
|  | | total (c) | -0.031 | 0.570 | -0.133 | 0.068 |
| SBP | | a | -0.104 | 0.847 | -0.174 | 0.124 |
|  | | b | 0.149 | *0.007* | **0.030** | **0.267** |
|  | | c’ | 0.014 | 0.808 | -0.102 | 0.111 |
|  | | ab | -0.002 | 0.847 | -0.033 | 0.018 |
|  | | total (c) | 0.011 | 0.841 | -0.113 | 0.111 |
| DBP | | a | -0.037 | 0.610 | -0.161 | 0.088 |
|  | | b | 0.147 | *0.008* | **0.023** | **0.262** |
|  | | c’ | -0.055 | 0.312 | -0.169 | 0.042 |
|  | | ab | -0.005 | 0.616 | -0.036 | 0.010 |
|  | | total (c) | -0.061 | 0.275 | -0.172 | 0.042 |
| waist | | a | -0.066 | 0.355 | -0.208 | 0.090 |
|  | | b | 0.148 | *0.007* | **0.031** | **0.267** |
|  | | c’ | -0.010 | 0.858 | -0.109 | 0.090 |
|  | | ab | -0.010 | 0.382 | -0.045 | 0.009 |
|  | | total (c) | -0.020 | 0.725 | -0.122 | 0.088 |
| glucose | | a | -0.060 | 0.419 | -0.184 | 0.104 |
|  | | b | 0.157 | *0.005* | **0.039** | **0.274** |
|  | | c’ | -0.012 | 0.827 | -0.110 | 0.110 |
|  | | ab | -0.009 | 0.437 | -0.039 | 0.012 |
|  | | total (c) | -0.022 | 0.705 | -0.126 | 0.108 |
| LDL | | a | -0.007 | 0.925 | -0.151 | 0.113 |
|  | | b | 0.140 | *0.012* | **0.019** | **0.262** |
|  | | c’ | -0.012 | 0.837 | -0.112 | 0.094 |
|  | | ab | -0.0009 | 0.925 | -0.030 | 0.019 |
|  | | total (c) | -0.013 | 0.826 | -0.114 | 0.095 |
| vLDL | | a | -0.119 | 0.103 | -0.273 | 0.026 |
|  | | b | 0.135 | *0.017* | **0.010** | **0.250** |
|  | | c’ | -0.047 | 0.404 | -0.191 | 0.080 |
|  | | ab | -0.016 | 0.178 | -0.057 | 0.001 |
|  | | total (c) | -0.063 | 0.267 | -0.207 | 0.074 |
| hemoglobinA1C | | a | 0.069 | 0.356 | -0.084 | 0.228 |
|  | | b | 0.139 | *0.013* | **0.012** | **0.255** |
|  | | c’ | 0.042 | 0.455 | -0.076 | 0.147 |
|  | | ab | 0.010 | 0.387 | -0.010 | 0.042 |
|  | | total (c) | 0.052 | 0.367 | -0.066 | 0158 |
| Plasma IL-6 | | a | -0.055 | 0.456 | -0.180 | 0.094 |
|  | | b | 0.142 | *0.011* | **0.022** | **0.263** |
|  | | c’ | -0.042 | 0.454 | -0.139 | 0.069 |
|  | | ab | -0.008 | 0.474 | -0.038 | 0.009 |
|  | | total (c) | -0.050 | 0.382 | -0.151 | 0.065 |
| Mean IMT | | a | 0.079 | 0.848 | -0.078 | 0.248 |
|  | | b | 0.141 | *0.012* | **0.017** | **0.258** |
|  | | c’ | 0.014 | 0.842 | -0.125 | 0.138 |
|  | | ab | 0.011 | 0.422 | -0.009 | 0.051 |
|  | | total (c) | 0.025 | 0.727 | -0.113 | 0.150 |
| Mean HR | | a | 0.001 | 0.988 | -0.134 | 0.134 |
|  | | b | 0.149 | *0.007* | **0.021** | **0.259** |
|  | | c’ | 0.052 | 0.351 | -0.060 | 0.157 |
|  | | ab | 0.0001 | 0.998 | -0.021 | 0.025 |
|  | | total (c) | 0.052 | 0.359 | -0.061 | 0.159 |
| PEP | | a | 0.008 | 0.906 | -0.144 | 0.145 |
|  | | b | 0.149 | *0.007* | **0.027** | **0.266** |
|  | | c’ | -0.060 | 0.264 | -0.153 | 0.046 |
|  | | ab | 0.001 | 0.906 | -0.021 | 0.028 |
|  | | total (c) | -0.059 | 0.284 | -0.162 | 0.048 |
| VOP % | | a | 0.079 | 0.276 | -0.037 | 0.203 |
|  | | b | 0.152 | *0.006* | **0.029** | **0.262** |
|  | | c’ | 0.002 | 0.977 | -0.120 | 0.120 |
|  | | ab | 0.012 | 0.312 | -0.003 | 0.042 |
|  | | total (c) | 0.014 | 0.808 | -0.104 | 0.129 |
| PWV | | a | 0.086 | 0.235 | -0.045 | 0.214 |
|  | | b | 0.151 | *0.006* | **0.033** | **0.272** |
|  | | c’ | -0.026 | 0.644 | -0.144 | 0.101 |
|  | | ab | 0.013 | 0.276 | -0.004 | 0.046 |
|  | | total (c) | -0.013 | 0.822 | -0.139 | 0.114 |
| SBP_SD | | a | 0.036 | 0.621 | -0.100 | 0.176 |
|  | | b | 0.149 | *0.007* | **0.028** | **0.262** |
|  | | c’ | -0.0005 | 0.991 | -0.142 | 0.112 |
|  | | ab | 0.005 | 0.627 | -0.013 | 0.036 |
|  | | total (c) | 0.005 | 0.993 | -0.130 | 0.036 |
| LF_BPV | | a | 0.009 | 0.899 | -0.139 | 0.153 |
|  | | b | 0.149 | *0.007* | **0.033** | **0.272** |
|  | | c’ | 0.069 | 0.204 | -0.028 | 0.167 |
|  | | ab | 0.001 | 0.899 | -0.021 | 0.028 |
|  | | total (c) | 0.071 | 0.204 | -0.026 | 0.163 |

| **Right** | label | estimated | p-value | CI. lower | CI. upper |
| --- | --- | --- | --- | --- | --- |
| Plasma VCAM | a | -0.258 | *2x10^-4^* | **-0.368** | **-0.140** |
|  | b | 0.137 | *0.020* | **0.006** | **0.271** |
|  | c’ | 0.069 | 0.232 | -0.037 | 0.166 |
|  | ab | -0.035 | *0.048* | **-0.077** | **-0.004** |
|  | total (c) | 0.034 | 0.551 | -0.070 | 0.123 |
| Plasma ICAM | a | -0.159 | *0.025* | **-0.300** | **-0.031** |
|  | b | 0.119 | *0.039* | -0.014 | 0.253 |
|  | c’ | 7.4x10^-4^ | 0.989 | -0.101 | 0.087 |
|  | ab | -0.019 | 0.129 | -0.060 | -2.7x10^-4^ |
|  | total (c) | -0.018 | 0.749 | -0.118 | 0.067 |
| SDNN | a | 0.156 | *0.032* | **0.031** | **0.316** |
|  | b | 0.130 | *0.022* | -0.001 | 0.263 |
|  | c’ | -0.064 | 0.264 | -0.179 | 0.031 |
|  | ab | 0.020 | 0.118 | **0.002** | **0.059** |
|  | total (c) | -0.044 | 0.445 | -0.148 | 0.048 |
| HF HRV | a | 0.171 | *0.020* | **0.039** | **0.308** |
|  | b | 0.123 | *0.031* | -0.013 | 0.257 |
|  | c’ | -0.017 | 0.768 | -0.143 | 0.097 |
|  | ab | 0.021 | 0.114 | 9.1x10^-4^ | 0.059 |
|  | total (c) | 0.004 | 0.949 | -0.111 | 0.113 |
| Mean BRS | a | 0.183 | *0.010* | **0.067** | **0.313** |
|  | b | 0.130 | *0.023* | -4.5x10^-4^ | 0.267 |
|  | c’ | -0.053 | 0.352 | -0.171 | 0.056 |
|  | ab | 0.024 | 0.089 | **0.003** | **0.059** |
|  | total (c) | -0.029 | 0.607 | -0.140 | 0.076 |
| Insulin | a | -0.130 | 0.071 | -0.267 | 0.034 |
|  | b | 0.115 | *0.044* | -0.003 | 0.258 |
|  | c’ | -0.047 | 0.403 | -0.051 | 0.054 |
|  | ab | -0.015 | 0.179 | -0.063 | 0.002 |
|  | total (c) | -0.062 | 0.273 | -0.162 | 0.044 |
| CO | a | 0.071 | 0.319 | -0.052 | 0.214 |
|  | b | 0.121 | *0.031* | -0.007 | 0.255 |
|  | c’ | -0.009 | 0.868 | -0.109 | 0.112 |
|  | ab | 0.009 | 0.366 | -0.003 | 0.040 |
|  | total (c) | -0.0005 | 0.992 | -0.100 | 0.120 |
| BMI | a | -0.099 | 0.166 | -0.238 | 0.074 |
|  | b | 0.114 | *0.042* | -0.014 | 0.251 |
|  | c’ | -0.063 | 0.253 | -0.161 | 0.036 |
|  | ab | -0.011 | 0.252 | -0.053 | 0.004 |
|  | total (c) | -0.075 | 0.181 | -0.169 | 0.025 |
| SBP | a | -0.031 | 0.675 | -0.183 | 0.118 |
|  | b | 0.121 | *0.031* | -0.004 | 0.259 |
|  | c’ | 0.018 | 0.758 | -0.108 | 0.136 |
|  | ab | -0.004 | 0.680 | -0.034 | 0.012 |
|  | total (c) | 0.014 | 0.811 | -0.115 | 0.135 |
| DBP | a | -0.080 | 0.265 | -0.218 | 0.046 |
|  | b | 0.116 | *0.039* | -0.018 | 0.246 |
|  | c’ | -0.056 | 0.320 | -0.177 | 0.061 |
|  | ab | -0.009 | 0.326 | -0.043 | 0.003 |
|  | total (c) | -0.065 | 0.249 | -0.186 | 0.054 |
| waist | a | -0.102 | 0.151 | -0.239 | 0.032 |
|  | b | 0.116 | *0.039* | -0.004 | 0.249 |
|  | c’ | -0.043 | 0.440 | -0.147 | 0.045 |
|  | ab | -0.012 | 0.239 | -0.051 | 0.001 |
|  | total (c) | -0.055 | 0.328 | -0.164 | 0.032 |
| glucose | a | -0.067 | 0.364 | -0.199 | 0.099 |
|  | b | 0.117 | *0.039* | -0.013 | 0.246 |
|  | c’ | -0.061 | 0.291 | -0.159 | 0.056 |
|  | ab | -0.008 | 0.406 | -0.039 | 0.007 |
|  | total (c) | -0.068 | 0.238 | -0.162 | 0.052 |
| LDL | a | 0.037 | 0.617 | -0.113 | 0.189 |
|  | b | 0.120 | *0.033* | -0.010 | 0.258 |
|  | c’ | -0.020 | 0.727 | -0.123 | 0.089 |
|  | ab | 0.004 | 0.626 | -0.011 | 0.035 |
|  | total (c) | -0.016 | 0.788 | -0.117 | 0.097 |
| vLDL | a | -0.100 | 0.171 | -0.267 | 0.057 |
|  | b | 0.112 | *0.048* | -0.014 | 0.250 |
|  | c’ | -0.077 | 0.173 | -0.230 | 0.057 |
|  | ab | -0.011 | 0.260 | -0.048 | 0.003 |
|  | total (c) | -0.089 | 0.121 | -0.244 | 0.056 |
| hemoglobinA1C | a | 0.095 | 0.200 | -0.059 | 0.248 |
|  | b | 0.122 | *0.031* | -0.008 | 0.256 |
|  | c’ | -0.040 | 0.487 | -0.168 | 0.072 |
|  | ab | 0.012 | 0.271 | -0.004 | 0.048 |
|  | total (c) | -0.029 | 0.624 | -0.159 | 0.082 |
| Plasma IL-6 | a | -0.056 | 0.441 | -0.192 | 0.102 |
|  | b | 0.117 | *0.039* | -0.019 | 0.246 |
|  | c’ | -0.049 | 0.392 | -0.187 | 0.069 |
|  | ab | -0.007 | 0.470 | -0.043 | 0.007 |
|  | total (c) | -0.055 | 0.336 | -0.192 | 0.065 |
| Mean IMT | a | 0.040 | 0.663 | -0.119 | 0.195 |
|  | b | 0.122 | *0.030* | -0.009 | 0.256 |
|  | c’ | -0.108 | 0.126 | -0.220 | 0.035 |
|  | ab | 0.005 | 0.669 | -0.012 | 0.036 |
|  | total (c) | -0.103 | 0.148 | -0.218 | 0.038 |
| Mean HR | a | -0.029 | 0.690 | -0.145 | 0.107 |
|  | b | 0.121 | *0.031* | -0.009 | 0.253 |
|  | c’ | 0.038 | 0.502 | -0.072 | 0.141 |
|  | ab | -0.004 | 0.695 | -0.028 | 0.011 |
|  | total (c) | 0.035 | 0.547 | -0.072 | 0.137 |
| PEP | a | -0.031 | 0.659 | -0.190 | 0.094 |
|  | b | 0.121 | *0.032* | -0.017 | 0.254 |
|  | c’ | 0.001 | 0.979 | -0.089 | 0.114 |
|  | ab | -0.004 | 0.665 | -0.033 | 0.011 |
|  | total (c) | -0.002 | 0.967 | -0.097 | 0.116 |
| VOP % | a | 0.073 | 0.315 | -0.113 | 0.104 |
|  | b | 0.122 | *0.032* | -0.015 | 0.267 |
|  | c’ | -0.010 | 0.861 | -0.113 | 0.104 |
|  | ab | 0.009 | 0.363 | -0.003 | 0.044 |
|  | total (c) | -0.001 | 0.985 | -0.109 | 0.112 |
| PWV | a | 0.032 | 0.661 | -0.105 | 0.171 |
|  | b | 0.120 | *0.032* | -0.009 | 0.255 |
|  | c’ | 0.019 | 0.731 | -0.090 | 0.144 |
|  | ab | 0.004 | 0.668 | -0.011 | 0.033 |
|  | total (c) | 0.023 | 0.684 | -0.088 | 0.151 |
| SBP_SD | a | 0.013 | 0.863 | -0.105 | 0.143 |
|  | b | 0.120 | *0.034* | -0.009 | 0.255 |
|  | c’ | 0.047 | 0.401 | -0.102 | 0.177 |
|  | ab | 0.002 | 0.864 | -0.011 | 0.027 |
|  | total (c) | 0.049 | 0.392 | -0.099 | 0.176 |
| LF_BPV | a | 0.009 | 0.900 | -0.124 | 0.164 |
|  | b | 0.120 | *0.034* | -0.006 | 0.257 |
|  | c’ | 0.026 | 0.645 | -0.097 | 0.149 |
|  | ab | 0.001 | 0.900 | -0.014 | 0.029 |
|  | total (c) | 0.027 | 0.635 | -0.096 | 0.148 |

**Supplementary table 2**. Mediation model for relationships between systemic variable and hippocampal volume through hippocampal supply vessel volume

| **Both** | label | estimated | p-value | CI. lower | CI. upper |
| --- | --- | --- | --- | --- | --- |
| Plasma VCAM | a | 0.020 | 0.757 | -0.107 | 0.168 |
|  | b | 0.082 | 0.169 | -0.055 | 0.212 |
|  | c’ | 0.023 | 0.671 | -0.093 | 0.115 |
|  | ab | 0.002 | 0.763 | -0.007 | 0.029 |
|  | total (c) | 0.024 | 0.650 | -0.086 | 0.114 |
| Plasma ICAM | a | -0.005 | 0.942 | -0.141 | 0.141 |
|  | b | 0.082 | 0.166 | -0.053 | 0.207 |
|  | c’ | 0.006 | 0.915 | -0.086 | 0.098 |
|  | ab | -0.0003 | 0.942 | -0.021 | 0.014 |
|  | total (c) | 0.005 | 0.921 | -0.086 | 0.098 |
| SDNN | a | 0.084 | 0.156 | -0.051 | 0.214 |
|  | b | -0.054 | 0.428 | -0.153 | 0.078 |
|  | c’ | 0.005 | 0.925 | -0.087 | 0.085 |
|  | ab | -0.004 | 0.489 | -0.028 | 0.004 |
|  | total (c) | 0.0006 | 0.990 | -0.090 | 0.083 |
| HF HRV | a | -0.009 | 0.890 | -0.130 | 0.115 |
|  | b | 0.083 | 0.156 | -0.041 | 0.225 |
|  | c’ | 0.022 | 0.696 | -0.079 | 0.125 |
|  | ab | -0.0007 | 0.890 | -0.022 | 0.010 |
|  | total (c) | 0.021 | 0.708 | -0.077 | 0.124 |
| Mean BRS | a | 0.061 | 0.357 | -0.058 | 0.163 |
|  | b | 0.084 | 0.157 | -0.052 | 0.220 |
|  | c’ | -0.002 | 0.975 | -0.093 | 0.091 |
|  | ab | 0.005 | 0.440 | -0.003 | 0.030 |
|  | total (c) | 0.003 | 0.949 | -0.090 | 0.091 |
| Insulin | a | 0.143 | *0.030* | **0.013** | **0.291** |
|  | b | 0.088 | 0.137 | -0.045 | 0.220 |
|  | c’ | -0.053 | 0.329 | -0.154 | 0.052 |
|  | ab | 0.013 | 0.220 | -0.003 | 0.050 |
|  | total (c) | -0.041 | 0.454 | -0.142 | 0.062 |
| CO | a | -0.098 | 0.131 | -0.202 | 0.017 |
|  | b | 0.082 | 0.164 | -0.043 | 0.207 |
|  | c’ | 0.035 | 0.513 | -0.060 | 0.145 |
|  | ab | -0.008 | 0.306 | -0.035 | 0.003 |
|  | total (c) | 0.027 | 0.615 | -0.065 | 0.135 |
| BMI | a | -0.035 | 0.597 | -0.177 | 0.104 |
|  | b | 0.076 | 0.195 | -0.050 | 0.209 |
|  | c’ | -0.050 | 0.350 | -0.138 | 0.047 |
|  | ab | -0.003 | 0.624 | -0.029 | 0.006 |
|  | total (c) | -0.052 | 0.327 | -0.143 | 0.041 |
| SBP | a | 0.008 | 0.910 | -0.119 | 0.131 |
|  | b | 0.078 | 0.185 | -0.053 | 0.204 |
|  | c’ | 0.013 | 0.818 | -0.109 | 0.124 |
|  | ab | 0.0005 | 0.911 | -0.010 | 0.017 |
|  | total (c) | 0.013 | 0.810 | -0.105 | 0.125 |
| DBP | a | -0.049 | 0.457 | -0.168 | 0.065 |
|  | b | 0.074 | 0.204 | -0.052 | 0.205 |
|  | c’ | -0.062 | 0.248 | -0.168 | 0.049 |
|  | ab | -0.004 | 0.521 | -0.032 | 0.003 |
|  | total (c) | -0.065 | 0.223 | -0.174 | 0.044 |
| waist | a | -0.011 | 0.862 | -0.152 | 0.136 |
|  | b | 0.077 | 0.186 | -0.055 | 0.204 |
|  | c’ | -0.035 | 0.516 | -0.131 | 0.061 |
|  | ab | -0.0008 | 0.863 | -0.023 | 0.010 |
|  | total (c) | -0.035 | 0.508 | -0.130 | 0.059 |
| glucose | a | 0.030 | 0.663 | -0.109 | 0.209 |
|  | b | 0.081 | 0.169 | -0.056 | 0.209 |
|  | c’ | -0.047 | 0.388 | -0.138 | 0.074 |
|  | ab | 0.002 | 0.678 | -0.007 | 0.032 |
|  | total (c) | -0.045 | 0.414 | -0.318 | 0.075 |
| LDL | a | 0.032 | 0.639 | -0.101 | 0.166 |
|  | b | 0.073 | 0.216 | -0.055 | 0.208 |
|  | c’ | -0.018 | 0.749 | -0.127 | 0.080 |
|  | ab | 0.002 | 0.661 | -0.005 | 0.026 |
|  | total (c) | -0.015 | 0.782 | -0.127 | 0.081 |
| vLDL | a | 0.0002 | 0.997 | -0.167 | 0.168 |
|  | b | 0.056 | 0.339 | -0.074 | 0.186 |
|  | c’ | -0.091 | 0.088 | -0.205 | 0.023 |
|  | ab | 0.0001 | 0.997 | -0.017 | 0.018 |
|  | total (c) | -0.091 | 0.088 | -0.204 | 0.022 |
| hemoglobinA1C | a | -0.002 | 0.978 | -0.137 | 0.122 |
|  | b | 0.077 | 0.191 | -0.058 | 0.203 |
|  | c’ | 0.014 | 0.807 | -0.103 | 0.126 |
|  | ab | -0.0001 | 0.978 | -0.017 | 0.012 |
|  | total (c) | 0.013 | 0.810 | -0.107 | 0.126 |
| Plasma IL-6 | a | 0.011 | 0.874 | -0.106 | 0.132 |
|  | b | 0.077 | 0.193 | -0.059 | 0.202 |
|  | c’ | -0.055 | 0.314 | -0.161 | 0.051 |
|  | ab | 0.0008 | 0.875 | -0.008 | 0.019 |
|  | total (c) | -0.054 | 0.324 | -0.162 | 0.051 |
| Mean IMT | a | 0.017 | 0.843 | -0.142 | 0.210 |
|  | b | 0.085 | 0.149 | -0.048 | 0.223 |
|  | c’ | -0.075 | 0.267 | -0.195 | 0.074 |
|  | ab | 0.001 | 0.844 | -0.012 | 0.035 |
|  | total (c) | -0.074 | 0.279 | -0.194 | 0.078 |
| Mean HR | a | 0.080 | 0.234 | -0.052 | 0.202 |
|  | b | 0.080 | 0.176 | -0.057 | 0.203 |
|  | c’ | 0.036 | 0.509 | -0.066 | 0.130 |
|  | ab | 0.006 | 0.371 | -0.004 | 0.035 |
|  | total (c) | 0.042 | 0.438 | -0.058 | 0.134 |
| PEP | a | -0.055 | 0.401 | -0.184 | 0.070 |
|  | b | 0.076 | 0.195 | -0.046 | 0.213 |
|  | c’ | -0.028 | 0.602 | -0.118 | 0.074 |
|  | ab | -0.004 | 0.481 | -0.028 | 0.004 |
|  | total (c) | -0.032 | 0.549 | -0.124 | 0.074 |
| VOP % | a | -0.095 | 0.150 | -0.230 | 0.024 |
|  | b | 0.076 | 0.199 | -0.053 | 0.211 |
|  | c’ | 0.007 | 0.890 | -0.097 | 0.125 |
|  | ab | -0.007 | 0.338 | -0.037 | 0.003 |
|  | total (c) | 0.0002 | 0.996 | -0.102 | 0.115 |
| PWV | a | -0.019 | 0.780 | -0.142 | 0.114 |
|  | b | 0.078 | 0.184 | -0.045 | 0.206 |
|  | c’ | 0.0002 | 1.000 | -0.119 | 0.120 |
|  | ab | -0.001 | 0.785 | -0.022 | 0.008 |
|  | total (c) | -0.001 | 0.979 | -0.117 | 0.121 |
| SBP_SD | a | -0.088 | 0.183 | -0.197 | 0.043 |
|  | b | 0.088 | 0.141 | -0.037 | 0.230 |
|  | c’ | 0.034 | 0.528 | -0.104 | 0.145 |
|  | ab | -0.008 | 0.324 | -0.039 | 0.003 |
|  | total (c) | 0.027 | 0.625 | -0.108 | 0.138 |
| LF_BPV | a | -0.013 | 0.844 | -0.125 | 0.122 |
|  | b | 0.085 | 0.152 | -0.049 | 0.220 |
|  | c’ | 0.051 | 0.340 | -0.061 | 0.159 |
|  | ab | -0.001 | 0.846 | -0.021 | 0.010 |
|  | total (c) | 0.050 | 0.353 | -0.059 | 0.160 |

| **Left** | label | estimated | p-value | CI. lower | CI. upper |
| --- | --- | --- | --- | --- | --- |
| Plasma VCAM | a | 0.067 | 0.310 | -0.063 | 0.213 |
|  | b | 0.087 | 0.154 | -0.032 | 0.215 |
|  | c’ | 0.007 | 0.902 | -0.126 | 0.104 |
|  | ab | 0.006 | 0.408 | -0.003 | 0.040 |
|  | total (c) | 0.013 | 0.820 | -0.108 | 0.106 |
| Plasma ICAM | a | -0.023 | 0.725 | -0.169 | 0.133 |
|  | b | 0.089 | 0.146 | -0.035 | 0.209 |
|  | c’ | 0.031 | 0.578 | -0.064 | 0.137 |
|  | ab | -0.002 | 0.732 | -0.029 | 0.009 |
|  | total (c) | 0.029 | 0.606 | -0.067 | 0.134 |
| SDNN | a | -0.040 | 0.551 | -0.155 | 0.091 |
|  | b | 0.099 | 0.100 | -0.023 | 0.221 |
|  | c’ | 0.050 | 0.374 | -0.037 | 0.146 |
|  | ab | -0.004 | 0.575 | -0.025 | 0.006 |
|  | total (c) | 0.046 | 0.417 | -0.044 | 0.147 |
| HF HRV | a | -0.012 | 0.858 | -0.136 | 0.100 |
|  | b | 0.098 | 0.106 | -0.027 | 0.224 |
|  | c’ | 0.037 | 0.511 | -0.063 | 0.144 |
|  | ab | -0.001 | 0.859 | -0.021 | 0.009 |
|  | total (c) | 0.036 | 0.527 | -0.064 | 0.142 |
| Mean BRS | a | 0.078 | 0.100 | -0.031 | 0.0009 |
|  | b | 0.095 | 0.122 | -0.029 | 0.219 |
|  | c’ | 0.029 | 0.604 | -0.057 | 0.123 |
|  | ab | 0.007 | 0.346 | -0.002 | 0.034 |
|  | total (c) | 0.036 | 0.515 | -0.053 | 0.129 |
| Insulin | a | 0.134 | *0.044* | -0.0005 | 0.277 |
|  | b | 0.097 | 0.111 | -0.026 | 0.227 |
|  | c’ | -0.027 | 0.625 | -0.131 | 0.085 |
|  | ab | 0.013 | 0.211 | -0.001 | 0.050 |
|  | total (c) | -0.014 | 0.796 | -0.118 | 0.097 |
| CO | a | -0.051 | 0.436 | -0.148 | 0.067 |
|  | b | 0.094 | 0.118 | -0.028 | 0.214 |
|  | c’ | 0.057 | 0.298 | -0.034 | 0.172 |
|  | ab | -0.005 | 0.485 | -0.025 | 0.003 |
|  | total (c) | 0.052 | 0.343 | -0.037 | 0.164 |
| BMI | a | -0.012 | 0.860 | -0.160 | 0.142 |
|  | b | 0.090 | 0.134 | -0.041 | 0.200 |
|  | c’ | -0.023 | 0.668 | -0.112 | 0.078 |
|  | ab | -0.001 | 0.861 | -0.021 | 0.013 |
|  | total (c) | -0.024 | 0.656 | -0.115 | 0.075 |
| SBP | a | 0.003 | 0.960 | -0.136 | 0.122 |
|  | b | 0.090 | 0.133 | -0.031 | 0.211 |
|  | c’ | 0.011 | 0.845 | -0.111 | 0.116 |
|  | ab | 0.0003 | 0.960 | -0.015 | 0.017 |
|  | total (c) | 0.011 | 0.842 | -0.113 | 0.116 |
| DBP | a | -0.019 | 0.771 | -0.140 | 0.098 |
|  | b | 0.089 | 0.137 | -0.030 | 0.215 |
|  | c’ | -0.058 | 0.293 | -0.161 | 0.037 |
|  | ab | -0.002 | 0.775 | -0.021 | 0.008 |
|  | total (c) | -0.059 | 0.281 | -0.163 | 0.037 |
| waist | a | 0.003 | 0.962 | -0.147 | 0.149 |
|  | b | 0.090 | 0.132 | -0.034 | 0.210 |
|  | c’ | -0.013 | 0.819 | -0.111 | 0.085 |
|  | ab | 0.0002 | 0.962 | -0.015 | 0.020 |
|  | total (c) | -0.012 | 0.824 | -0.114 | 0.084 |
| glucose | a | 0.084 | 0.217 | -0.059 | 0.269 |
|  | b | 0.095 | 0.116 | -0.032 | 0.218 |
|  | c’ | -0.025 | 0.662 | -0.124 | 0.096 |
|  | ab | 0.008 | 0.332 | -0.004 | 0.045 |
|  | total (c) | -0.017 | 0.768 | -0.120 | 0.102 |
| LDL | a | -0.017 | 0.803 | -0.157 | 0.115 |
|  | b | 0.081 | 0.180 | -0.036 | 0.210 |
|  | c’ | -0.012 | 0.831 | -0.118 | 0.091 |
|  | ab | -0.001 | 0.807 | -0.025 | 0.008 |
|  | total (c) | -0.013 | 0.813 | -0.117 | 0.088 |
| vLDL | a | -0.036 | 0.590 | -0.178 | 0.121 |
|  | b | 0.053 | 0.378 | -0.067 | 0.178 |
|  | c’ | -0.051 | 0.354 | -0.163 | 0.054 |
|  | ab | -0.002 | 0.646 | -0.027 | 0.006 |
|  | total (c) | -0.053 | 0.337 | -0.161 | 0.051 |
| hemoglobinA1C | a | -0.012 | 0.859 | -0.139 | 0.127 |
|  | b | 0.090 | 0.136 | -0.027 | 0.211 |
|  | c’ | 0.056 | 0.324 | -0.058 | 0.169 |
|  | ab | -0.001 | 0.860 | -0.020 | 0.010 |
|  | total (c) | 0.055 | 0.337 | -0.060 | 0.170 |
| Plasma IL-6 | a | 0.009 | 0.890 | -0.109 | 0.135 |
|  | b | 0.084 | 0.165 | -0.032 | 0.210 |
|  | c’ | -0.048 | 0.390 | -0.150 | 0.057 |
|  | ab | 0.0007 | 0.890 | -0.010 | 0.019 |
|  | total (c) | -0.047 | 0.401 | -0.152 | 0.055 |
| Mean IMT | a | 0.009 | 0.912 | -0.161 | 0.227 |
|  | b | 0.096 | 0.114 | -0.022 | 0.224 |
|  | c’ | -0.038 | 0.592 | -0.172 | 0.111 |
|  | ab | 0.0008 | 0.912 | -0.018 | 0.032 |
|  | total (c) | -0.037 | 0.603 | -0.176 | 0.120 |
| Mean HR | a | 0.086 | 0.197 | -0.037 | 0.224 |
|  | b | 0.093 | 0.125 | -0.036 | 0.221 |
|  | c’ | 0.038 | 0.496 | -0.073 | 0.141 |
|  | ab | 0.008 | 0.323 | -0.002 | 0.040 |
|  | total (c) | 0.046 | 0.411 | -0.065 | 0.147 |
| PEP | a | -0.043 | 0.514 | -0.169 | 0.081 |
|  | b | 0.087 | 0.145 | -0.037 | 0.204 |
|  | c’ | -0.055 | 0.313 | -0.149 | 0.045 |
|  | ab | -0.004 | 0.551 | -0.028 | 0.006 |
|  | total (c) | -0.059 | 0.283 | -0.157 | 0.042 |
| VOP % | a | -0.104 | 0.116 | -0.239 | 0.019 |
|  | b | 0.089 | 0.142 | -0.039 | 0.213 |
|  | c’ | 0.011 | 0.846 | -0.108 | 0.136 |
|  | ab | -0.009 | 0.283 | -0.038 | 0.002 |
|  | total (c) | 0.002 | 0.978 | -0.115 | 0.124 |
| PWV | a | -0.008 | 0.901 | -0.143 | 0.121 |
|  | b | 0.090 | 0.134 | -0.028 | 0.219 |
|  | c’ | -0.025 | 0.647 | -0.142 | 0.097 |
|  | ab | -0.0007 | 0.901 | -0.022 | 0.011 |
|  | total (c) | -0.026 | 0.639 | -0.142 | 0.098 |
| SBP_SD | a | -0.080 | 0.224 | -0.196 | 0.058 |
|  | b | 0.098 | 0.109 | -0.039 | 0.221 |
|  | c’ | 0.009 | 0.867 | -0.110 | 0.115 |
|  | ab | -0.008 | 0.333 | -0.037 | 0.004 |
|  | total (c) | 0.001 | 0.980 | -0.115 | 0.105 |
| LF_BPV | a | -0.023 | 0.725 | -0.137 | 0.100 |
|  | b | 0.099 | 0.102 | -0.028 | 0.229 |
|  | c’ | 0.070 | 0.197 | -0.027 | 0.167 |
|  | ab | -0.002 | 0.731 | -0.024 | 0.008 |
|  | total (c) | 0.068 | 0.215 | -0.027 | 0.161 |

| **Right** | label | estimated | p-value | CI. lower | CI. upper |
| --- | --- | --- | --- | --- | --- |
| Plasma VCAM | a | -0.022 | 0.747 | -0.154 | 0.123 |
|  | b | 0.064 | 0.295 | -0.066 | 0.209 |
|  | c’ | 0.035 | 0.534 | -0.075 | 0.126 |
|  | ab | -0.001 | 0.758 | -0.027 | 0.007 |
|  | total (c) | 0.034 | 0.551 | -0.075 | 0.121 |
| Plasma ICAM | a | 0.011 | 0.866 | -0.115 | 0.151 |
|  | b | 0.063 | 0.301 | -0.074 | 0.202 |
|  | c’ | -0.019 | 0.738 | -0.125 | 0.074 |
|  | ab | 0.0007 | 0.868 | -0.009 | 0.022 |
|  | total (c) | -0.018 | 0.749 | -0.118 | 0.073 |
| SDNN | a | -0.057 | 0.408 | -0.162 | 0.061 |
|  | b | 0.055 | 0.359 | -0.075 | 0.188 |
|  | c’ | -0.041 | 0.478 | -0.141 | 0.050 |
|  | ab | -0.003 | 0.539 | -0.027 | 0.003 |
|  | total (c) | -0.044 | 0.445 | -0.147 | 0.048 |
| HF HRV | a | -0.006 | 0.933 | -0.138 | 0.135 |
|  | b | 0.058 | 0.337 | -0.078 | 0.197 |
|  | c’ | 0.004 | 0.944 | -0.116 | 0.110 |
|  | ab | -0.0003 | 0.934 | -0.017 | 0.010 |
|  | total (c) | 0.004 | 0.949 | -0.115 | 0.111 |
| Mean BRS | a | 0.038 | 0.578 | -0.087 | 0.160 |
|  | b | 0.060 | 0.327 | -0.075 | 0.194 |
|  | c’ | -0.031 | 0.579 | -0.139 | 0.074 |
|  | ab | 0.002 | 0.628 | -0.004 | 0.024 |
|  | total (c) | -0.029 | 0.607 | -0.137 | 0.077 |
| Insulin | a | 0.132 | 0.052 | -0.010 | 0.264 |
|  | b | 0.066 | 0.280 | -0.070 | 0.194 |
|  | c’ | -0.071 | 0.215 | -0.172 | 0.036 |
|  | ab | 0.009 | 0.345 | -0.006 | 0.040 |
|  | total (c) | -0.062 | 0.273 | -0.166 | 0.039 |
| CO | a | -0.125 | 0.060 | -0.238 | 0.002 |
|  | b | 0.056 | 0.358 | -0.079 | 0.196 |
|  | c’ | 0.006 | 0.910 | -0.097 | 0.127 |
|  | ab | -0.007 | 0.409 | -0.038 | 0.007 |
|  | total (c) | -0.0005 | 0.992 | -0.102 | 0.119 |
| BMI | a | -0.050 | 0.461 | -0.195 | 0.089 |
|  | b | 0.051 | 0.398 | -0.086 | 0.184 |
|  | c’ | -0.072 | 0.196 | -0.168 | 0.020 |
|  | ab | -0.003 | 0.579 | -0.032 | 0.005 |
|  | total (c) | -0.075 | 0.181 | -0.168 | 0.020 |
| SBP | a | 0.010 | 0.883 | -0.111 | 0.133 |
|  | b | 0.055 | 0.363 | -0.085 | 0.190 |
|  | c’ | 0.013 | 0.818 | -0.123 | 0.134 |
|  | ab | 0.0005 | 0.885 | -0.008 | 0.016 |
|  | total (c) | 0.014 | 0.811 | -0.121 | 0.134 |
| DBP | a | -0.068 | 0.316 | -0.185 | 0.046 |
|  | b | 0.050 | 0.405 | -0.086 | 0.180 |
|  | c’ | -0.061 | 0.275 | -0.182 | 0.059 |
|  | ab | -0.003 | 0.522 | -0.028 | 0.004 |
|  | total (c) | -0.065 | 0.249 | -0.184 | 0.058 |
| waist | a | -0.022 | 0.742 | -0.159 | 0.114 |
|  | b | 0.053 | 0.373 | -0.077 | 0.189 |
|  | c’ | -0.054 | 0.337 | -0.150 | 0.038 |
|  | ab | -0.001 | 0.757 | -0.023 | 0.006 |
|  | total (c) | -0.055 | 0.328 | -0.512 | 0.038 |
| glucose | a | -0.020 | 0.776 | -0.151 | 0.138 |
|  | b | 0.054 | 0.373 | -0.084 | 0.189 |
|  | c’ | -0.067 | 0.244 | -0.164 | 0.044 |
|  | ab | -0.001 | 0.786 | -0.022 | 0.008 |
|  | total (c) | -0.068 | 0.238 | -0.166 | 0.046 |
| LDL | a | 0.069 | 0.322 | -0.063 | 0.209 |
|  | b | 0.055 | 0.362 | -0.082 | 0.181 |
|  | c’ | -0.019 | 0.738 | -0.134 | 0.073 |
|  | ab | 0.004 | 0.502 | -0.004 | 0.029 |
|  | total (c) | -0.016 | 0.788 | -0.128 | 0.078 |
| vLDL | a | 0.031 | 0.656 | -0.124 | 0.211 |
|  | b | 0.052 | 0.390 | -0.087 | 0.187 |
|  | c’ | -0.122 | *0.031* | -0.247 | 0.006 |
|  | ab | 0.002 | 0.692 | -0.007 | 0.032 |
|  | total (c) | -0.120 | *0.033* | -0.248 | 0.005 |
| hemoglobinA1C | a | 0.007 | 0.920 | -0.131 | 0.153 |
|  | b | 0.054 | 0.374 | -0.079 | 0.193 |
|  | c’ | -0.029 | 0.619 | -0.151 | 0.088 |
|  | ab | 0.0003 | 0.921 | -0.010 | 0.017 |
|  | total (c) | -0.029 | 0.624 | -0.152 | 0.087 |
| Plasma IL-6 | a | 0.010 | 0.880 | -0.110 | 0.143 |
|  | b | 0.057 | 0.341 | -0.073 | 0.192 |
|  | c’ | -0.056 | 0.329 | -0.189 | 0.065 |
|  | ab | 0.0005 | 0.882 | -0.007 | 0.019 |
|  | total (c) | -0.055 | 0.336 | -0.192 | 0.065 |
| Mean IMT | a | 0.021 | 0.810 | -0.134 | 0.188 |
|  | b | 0.060 | 0.323 | -0.081 | 0.193 |
|  | c’ | -0.104 | 0.143 | -0.214 | 0.033 |
|  | ab | 0.001 | 0.816 | -0.008 | 0.029 |
|  | total (c) | -0.103 | 0.148 | -0.211 | 0.040 |
| Mean HR | a | 0.063 | 0.358 | -0.065 | 0.189 |
|  | b | 0.056 | 0.356 | -0.084 | 0.198 |
|  | c’ | 0.031 | 0.589 | -0.078 | 0.142 |
|  | ab | 0.004 | 0.515 | -0.004 | 0.030 |
|  | total (c) | 0.035 | 0.547 | -0.071 | 0.146 |
| PEP | a | -0.058 | 0.388 | -0.192 | 0.077 |
|  | b | 0.055 | 0.363 | -0.081 | 0.195 |
|  | c’ | 0.0008 | 0.988 | -0.102 | 0.111 |
|  | ab | -0.003 | 0.531 | -0.030 | 0.004 |
|  | total (c) | -0.002 | 0.967 | -0.107 | 0.110 |
| VOP % | a | -0.075 | 0.271 | -0.204 | 0.053 |
|  | b | 0.055 | 0.364 | -0.085 | 0.187 |
|  | c’ | 0.003 | 0.957 | -0.105 | 0.117 |
|  | ab | -0.004 | 0.484 | -0.030 | 0.005 |
|  | total (c) | -0.001 | 0.985 | -0.107 | 0.108 |
| PWV | a | -0.025 | 0.717 | -0.512 | 0.111 |
|  | b | 0.055 | 0.356 | -0.075 | 0.189 |
|  | c’ | 0.025 | 0.666 | -0.095 | 0.148 |
|  | ab | -0.001 | 0.736 | -0.025 | 0.006 |
|  | total (c) | 0.023 | 0.684 | -0.097 | 0.147 |
| SBP_SD | a | -0.082 | 0.226 | -0.194 | 0.040 |
|  | b | 0.063 | 0.299 | -0.078 | 0.204 |
|  | c’ | 0.054 | 0.344 | -0.089 | 0.184 |
|  | ab | -0.005 | 0.430 | -0.033 | 0.005 |
|  | total (c) | 0.049 | 0.392 | -0.095 | 0.183 |
| LF_BPV | a | -0.003 | 0.969 | -0.135 | 0.126 |
|  | b | 0.058 | 0.337 | -0.070 | 0.199 |
|  | c’ | 0.027 | 0.633 | -0.109 | 0.148 |
|  | ab | -0.0001 | 0.969 | -0.015 | 0.010 |
|  | total (c) | 0.027 | 0.635 | -0.108 | 0.148 |
